# Convergence Gerontology: Rethinking Translation in Research on Aging

**DOI:** 10.1093/geroni/igaa003

**Published:** 2020-03-07

**Authors:** Steven M Albert

**Affiliations:** Department of Behavioral and Community Health Sciences, Graduate School of Public Health, University of Pittsburgh, Pittsburgh, Pennsylvania

Translation in research on aging is the hallmark of *Innovation in Aging* (IA). Accordingly, a statement on translation, prominently placed in a blue box at the start of each article, is required of every article published. We reviewed the “translational significance” statements from the first 95 articles appearing *IA*. These are hard to categorize, considering the great range of topics that appear in the journal. The common denominator for the statements is a recommendation for a change in policy, practice, or simply the way we think about a problem based on recognition of greater complexity in the problem at hand. This is an excellent start but raises the larger question of how we should think about translation in gerontology. With 3 years of publishing experience under our belt, it is appropriate now to see how our field thinks about translation and perhaps to push the field in this direction.

## Defining Translation

Translation is usually limited to the potential for implementation: how likely research will change a policy, alter an environment, or support an intervention. Indeed, CDC asks investigators to detail “policy and environmental changes” emerging from funded research to assess translation. Typical phrases are “bringing to scale” or “disseminating results.” This approach seems too narrow for *IA* (and, indeed, gerontology) because much of our research is more basic. For example, consider the following kinds of research: quantifying the greater risk of disability with declines in gait speed, assessing the additional risk of nursing home placement associated with unmet ADL needs, assessing variation in stress among family caregivers of different ethnicities, and using the electronic health record to identify differences in patterns of care associated with a diagnosis of dementia. All carry translational significance but none immediately suggests a program to implement or a clinical intervention. These efforts are certainly useful for translation but represent more basic behavioral and social science. They do not offer immediate implementation, yet they are “translational” in that they bring together data, methods, and analyses to address a problem and suggest a new direction or approach.

If we think about translation this way, we can define translation as *enhancing the potential to solve a problem*. This is not the typical way we think about research translation. It is not the standard research pipeline of T1–T4 bench science to clinical application to policy change, or the research–evaluation–dissemination model. It is rather posing a problem in a way that speeds solutions.

Going a step further, the challenge of translation then becomes conceptualizing problems better. How do we do this? One productive approach is to bring disciplines together to address a problem, with the different methodologies and research designs each offers. This involves translating a research problem across disciplines. This challenge has recently been addressed through “convergence science.” As one influential review suggests, we need to “reimagine population health as convergence science” (Dzau & Balatbat, 2018). At a minimum, convergence science seeks multidisciplinary or transdisciplinary collaboration. Translation in gerontology could begin with translating a research problem for multidisciplinary investigation.

## Convergence Science

In thinking about convergence gerontology, let us start with a definition of convergence in mental health research. Following Eyre, Lavretsky, and Insel (2015), convergence involves integration of “biological, psychological, sociocultural, and environmental data.” They point to examples of convergence currently underway in mental health research, which are worth summarizing here. These include “continuous assessment of mental state using voice analytics, facial expression monitoring, actigraphy, and engagement of social networks to track depression or mania” and “electronic mental health interventions using personalized smartphone apps to deliver psychosocial treatments.”

Note that these efforts involve data and methodologies that cross disciplines. As pointed out elsewhere, this convergence forces us to think about mechanisms for mental illness in a different way (Albert & Ricci, 2020). To take an example, why should a smartphone app be more effective in addressing depression than traditional therapies? The app interface captures real-time data for tracking mood and function and can provide cognitive behavioral therapy tailored to particular daily challenges. Is its success due to this constant availability linked to challenging moments across the day? This feature would suggest that depression cannot be separated from social triggers and that therapy needs to be delivered on an as-needed basis. Or is the success of the app platform due to its ability to link therapy to other aspects of behavior tracked by the interface, such as an increased heart or breathing rate, which are often physical signs of an impending anxiety episode? This would suggest an alternative mechanism for the condition and other routes for therapy.

The convergence science producing the app, from engineering to psychiatry to human–machine interaction, leads us to recognize that depression is different in some ways from how mental health professionals typically think about it. This recognition should promote better problem solving. Translation across disciplines is a key step toward developing more standard translational pathways.

Switching to an example from public health, consider the following problem. In a typical city in the United States, approximately 15% of the residential land is vacant or abandoned (Branas et al., 2018; South, Hohl, Kondo, MacDonald, & Branas, 2018). These vacant plots are concentrated in low-income neighborhoods. Proximity to vacant lots and physical blight has been associated with crime, less outdoor physical activity, and poorer mental health (Kondo, Fluehr, McKeon, & Branas, 2018; Kondo, Morrison, et al., 2018). Vacant lots also increase the risk of environmental hazards, such as lead in soil from demolitions and flooding from storm runoff, which may be mixed with sewage backup. Vacant urban plots affect public health on every front, from psychological harm to environmental exposures. It stands to reason that a convergent approach will have better success in assessing the dimensions and dynamics of the problem.

One public health solution involves greening interventions. U.S. communities as different as Philadelphia, New Orleans, Detroit, and Youngstown have reported benefit from such efforts. Benefits have been demonstrated in both observational studies and randomized controlled trials. Areas targeted for greening have seen reductions in firearm violence relative to control areas (Moyer, MacDonald, Ridgeway, & Branas, 2018). In New Orleans, drug crimes per square mile decreased for residents living near remediated lots (Kondo, Morrison, et al., 2018). In Detroit, census tracts that completed 5+ demolitions of abandoned buildings saw reductions in firearm assaults. An observational study in Australia found that more total green space (tree canopy, in particular) was associated with a lower incidence of psychological distress (Astell-Burt & Feng, 2019).

Now consider this convergent public health perspective. In Pittsburgh, Pennsylvania, low-income communities are more likely to be located in flood zones or areas that experienced earlier mining extraction. Surveys suggest that 40% of homes in these communities experience a wet basement during flood events and perhaps 20% have sewer backups. These communities also have the highest incidence of GI illness involving CDC-reportable gastrointestinal (GI) pathogens (*salmonella*, *cryptosporidium*, *campylobacter*) in the city, with rates double the average of Pittsburgh as a whole.

With this information, a convergence science approach suggests that the problem is much larger and more complex. In addition to expertise from criminology, community psychiatry, and environmental science, we see that adequate understanding of the problem requires hydrogeologic expertise to map flood areas. We need the expertise of environmental engineering to correlate chemical/bacteriologic analysis of water samples with reports of GI symptoms (e.g., days with diarrhea) and community-level GI-related health care (from local hospital electronic health records). New hypotheses emerge: (1) Are GI symptoms in flood areas higher than in periphery areas? (2) Are GI symptoms most prevalent in households with flooding characterized by sewage backup? (3) Are GI pathogens more prominent in water samples with high levels of sewage backup? Most critically for our interests, we have a new outcome: Does greening remediation reduce community-level GI symptom burden?

This broad-scale convergence effort is inconceivable without the input of multiple disciplines and a wide range of methodologies, including hydrogeologic mapping, laboratory testing for bacteriology and contaminants, storm water sewage characterization using oxygen and nitrogen isotope ratios, address-based sampling, and community-level analysis of electronic health record (EHR) data.

## Convergence Gerontology

When we spend tax dollars on road building rather than on public transportation, we may affect the ability of older persons to maintain themselves in the community. The fact that the connection is not obvious does not negate its seriousness. (Lawton & Nahemow, 1973)

How can the convergence perspective make aging research more translational? One useful perspective is CDC’s *Healthy People 2020*, which proposes a social ecologic approach to the determinants of health. In the social ecologic perspective, health is affected by larger social, economic, and environmental factors. Interrelationships among these factors, including policymaking, social factors, health services, individual behavior, and biology and genetics, determine individual and population health. “Determinants of health reach beyond the boundaries of traditional health care and public health sectors; sectors such as education, housing, transportation, agriculture, and environment can be important allies in improving population health” (https://www.healthypeople.gov/2020/about/foundation-health-measures/Determinants-of-Health#biology%20and%20genetics).

This approach suggests the need for convergence in aging research. As Lawton and Nahemow (1973) suggest, spending tax dollars on road building rather than public transportation may affect the ability of older persons to maintain themselves independently in the community. We need to look at aging people and populations in this broader way to see the value of a more translational approach to aging research. A further implication of the CDC approach is the need to develop interventions that target multiple determinants of health. These are likely to be more effective than the one intervention–one problem approach we usually adopt.

**Figure 1.**
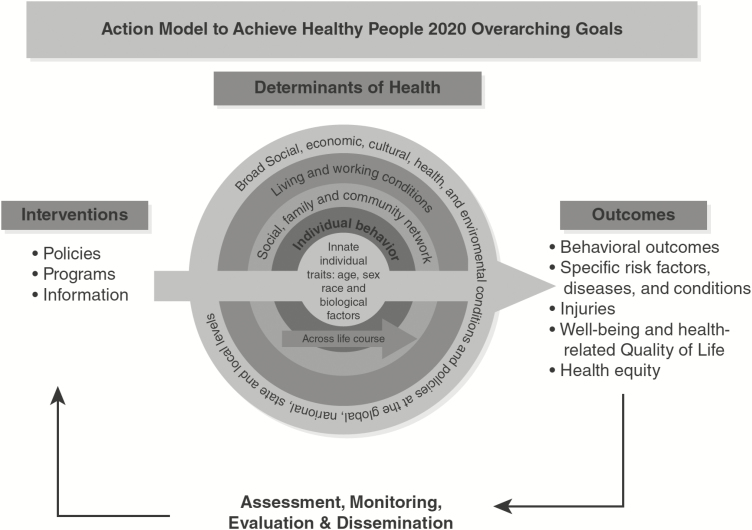
Action model to achieve Healthy People 2020 overarching goals. Reproduced from CDC (2010).

A challenge of this approach is to move beyond the typical social-ecologic models of concentric circles of influence, which imply dynamics but usually do not specify how determinants of health influence one other or how systems change over time. However, the CDC model specifies some of these relationships, which makes it useful for convergence and translational science in aging. The *Healthy People 2020* CDC model is reproduced as Figure 1. It suggests that effective interventions do not simply change outcomes, such as health behaviors or the prevalence of disability. Determinants of health, interventions, and outcomes are related in a causal loop, such that changes induced by an effective intervention also feedback to influence the determinants of health.

The challenge of convergence gerontology is to understand which determinant is affected by an intervention and how these determinants are themselves related. As an example, consider attempts to improve medication regimens among older people. Prescription drug use is ubiquitous in old age. Among adults aged 40–79, for example, NHANES data show that 69% used at least one prescription drug in the past 30 days and 22.4% at least five (Hales, Servais, Martin, & Kohen, 2019). Eighty-five percent of adults aged 60 and older use at least one prescription medication (Martin, Hales, Gu, & Ogden, 2019). The risks of such intensive prescription medication use are well known. For example, one large electronic medical record case–control study found that 10 of the 20 most commonly prescribed medications were associated with falls (Kuschel, Laflamme, & Möller, 2015). Medications affecting the central nervous system (opioids and antidepressants) carried the highest risk for fall injuries. Notably, 7 of the top 20 were cardiovascular medications, such as angiotensin converting enzyme inhibitors and selective calcium channel blockers, and these, by contrast, had a protective effect.

These studies are complicated because of confouding by indication: people who take more prescriptions have more medical conditions, which makes it difficult to attribute their greater risk of falls or other adverse outcomes to medications alone. However, in prior work with a large retiree database, we were able to show that retirees taking one or more potentially inappropriate medications were 1.8–1.9 times more likely to have a hospital admission in models that adjusted for age, gender, number of prescriptions overall, and aggregate disease severity. Risk of hospitalization also increased in a dose–response relationship according to the number of potentially inappropriate medications (Albert, Colombi, & Hanlon, 2010).

How then can we apply convergence gerontology to this challenge? It would be best to combine the expertise of pharmacoepidemiology, clinical pharmacy, medical informatics, evaluation science, and gerontology, at the least. It would also help to have a strong interventional framework to see whether oversight of medication regimens reduces adverse events in any way. One approach is to work with large administrative efforts to regulate prescribing, such as prescription benefit management programs or pharmaceutical subsidy programs for older adults. These programs control prescribing at the point of sale, for example, by checking for duplication and making it harder for patients to fill prescriptions for medications that are potentially inappropriate for older adults. To assess the effects of such programs, however, we need medical and health care utilization outcomes. Thus, we need to link prescription information with medical claims or electronic health records. We also need a comparison group, such as matched older adults with health claims who do not participate in the prescription management program.

The complexity of the study and need for different kinds of expertise is clear. Rather than a simple interrogation of the data to see whether a particular medication or therapeutic class is associated with any one adverse outcome, we need to examine evolving prescription regimens and their indications relative to patterns of evolving health care utilization. Is prescribing different in people with similar comorbidity profiles according to program participation? For example, are psychiatric medications or opioids less likely to be ordered or filled? Is the pattern of medical care different? The scale of these datasets and the many variables assessed may require machine learning techniques and collaboration with experts in biomedical informatics. Effective analysis of the dataset may need to draw on other tools as well, such as the Observational Health Data Sciences and Informatics (OHDSI) platform, to process drug codes and aggregate ICD diagnoses. This kind of convergence gerontology is essential if we are to understand why, for example, some older adults quickly return to stable community medication regimes after a hospital discharge while others do not. Convergence gerontology should allow a better understanding of the ecosystem of aging.

## Conclusions

We have argued that translation in aging research is most productively viewed from the perspective of convergence: bringing in expertise from different disciplines to rethink research challenges and apply different kinds of data and analytic tools. Translating a research problem across disciplines may speed solutions to the major challenges of aging. Two immediate tasks should push aging science in this direction:

- The Gerontological Society of America should consider convening a workgroup to think about systems science and social ecology in aging. How can we adapt the CDC model of determinants of health for aging? What data sources are missing? Which mechanisms linking levels in this social ecology are well understood and which not?
- To determine the additional translational benefit from convergence, funding agencies that support aging research should considering the NSF model of explicitly requiring disciplines to collaborate, as illustrated by its Long-Term Ecological Modeling program (LTER). Would such an approach shorten the time between research discovery and implementation in aging, which still remains unacceptably long?

*Innovation in Aging* will continue to pursue translation in aging science. We challenge aging researchers, policymakers, and clinical practitioners to adopt convergence as an approach to aging science. *IA* has already taken steps in this direction. A systems science approach to human capital (Morrow-Howell, Halvorsen, Hovmand, Lee, & Ballard, 2017) suggests new approaches to measuring engagement over the lifespan. Research on within-family differences involving parent–child dyads connects developmental psychological studies of children to the dynamics of the family in later life (Pillemer & Gilligan, 2018). IA authors have developed new models drawing on human factors and architectural research to separate aging with disability from aging into disability (Mitzner, Sanford, & Rogers, 2018). IA calls for papers have begun to push toward convergence perspectives, such as its special issue on translational caregiving research (Suitor, 2019).

To further this effort, *IA* will continue to promote special issues on cross-disciplinary approaches to major challenges in aging. We will also develop a call for papers that explicitly seeks joint authorships from diverse disciplines. We look forward to helping aging research become an increasing convergent science.
